# Supplementary material for: Infection Heterogeneity and Microbiota Differences in Chicks Infected by Salmonella enteritidis
Source: Microorganisms. 2021 Aug 11;9(8):1705. doi: 10.3390/microorganisms9081705 (PMC8399513; doi:10.3390/microorganisms9081705)
Supplement: Supplementary file 1 [file microorganisms-09-01705-s001.zip › microorganisms-1302799-supplementary.pdf]

Article

Infection heterogeneity and microbiota differences in chicks infected by *Salmonella enteritidis*

Shu Wu [1,2], Guanglei Cong [2], Qianyun Zhang [2], Hong Yao [2], Zhenxin Wang [2], Kelang Kang [1,2], Xi He [1,*], and Shourong Shi [2,3,4,*]

[1] College of Animal Science and Technology, Hunan Agricultural University, Changsha 410128, China;
wushu223759@163.com (S.W.); kangkelang@126.com (K.K.)

[2] Department of Feed and Nutrition, Poultry Institute, Chinese Academy of Agricultural Sciences,
Yangzhou 225125, China; MZ120181027@yzu.edu.cn (G.C.); 17858765721@163.com (Q.Z.); Ravenpeach@163.com (H.Y.); 17863856330@163.com (Z.W.)

[3] Institute of Effective Evaluation of Feed and Feed Additive (Poultry Institute), Ministry of Agriculture, Yangzhou 225125, China

[4] Jiangsu Co-Innovation Center for the Prevention and Control of Important Animal Infectious Disease and Zoonose, Yangzhou University, Yangzhou 225009, China

* Correspondence: hexi111@126.com (X.H.); ssr236@163.com (S.S.)

Table S1. Tissue *Salmonella* loads of all slaughtered chicks (n = 48).

Chick number	Liver *Salmonella* loads (log_{10}CFU/g)	Spleen *Salmonella* loads (log_{10}CFU/g)	Sample grouping
35	3.301	5.134	Group R
28	3.380	5.048	
31	3.477	5.001	
48	3.256	4.997	
27	2.603	4.571	
5	0.000	4.544	
14	3.080	4.508	
8	2.904	4.466	
26	3.146	4.236	
3	2.303	4.049	
39	2.303	3.806	
42	0.000	3.764	
25	0.000	3.732	
33	0.000	3.623	
1	0.000	3.580	
47	2.303	3.447	
23	2.303	3.380	
29	0.000	3.380	
12	0.000	3.343	
18	0.000	3.301	
7	2.303	3.256	
17	0.000	3.256	
19	2.603	3.256	
37	0.000	3.204	
16	0.000	3.146	
22	0.000	3.146	
30	3.343	3.080	
46	2.303	3.080	
15	2.603	3.000	
41	2.303	3.000	
34	0.000	3.000	
40	0.000	2.904	
20	0.000	2.904	
6	2.303	2.779	
9	2.303	2.779	
21	2.303	2.779	
43	0.000	2.779	
44	0.000	2.779	
13	3.000	2.603	
32	2.303	2.603	

38	2.303	2.603	
10	2.303	2.603	
4	0.000	2.603	
2	0.000	2.603	Group S
11	0.000	2.603	
24	2.303	2.303	
36	2.303	2.303	
45	0.000	2.303	

Table S2. Details of sequencing data.

Sample name1	Effective reads	Good's coverage (%)	OTU number
R1	53040	0.997	601
R2	51735	0.997	663
R3	55739	0.999	368
R4	55698	0.997	601
R5	58617	0.997	543
R6	58629	0.998	538
R7	42192	0.996	1008
R8	56171	0.996	942
S1	56833	0.995	870
S2	60920	0.997	679
S3	49545	0.997	849
S4	49244	0.996	826
S5	54498	0.997	689
S6	45061	0.997	768
S7	43113	0.997	767
S8	66094	0.999	390

[1]R = the selected *S.enteritidis* resistant chicks; S = the selected *S.enteritidis* susceptible chicks.

Table S3. Alpha diversity analysis of cecal microbiota (n = 8).

	ACE	Chao1	PD_whole_tree	Shannon	Simpson
Group R	730.83±83.97	703.96±79.86	53.84±4.87	5.34±0.26	0.91±0.02
Group S	808.22±63.09	803.41±61.53	67.56±8.15	5.52±0.26	0.90±0.03
p-value	0.279	0.279	0.160	0.382	1.000

Group R = the selected *S.enteritidis* resistant chicks; Group S = the selected *S.enteritidis*-susceptible chicks. Differences between two groups were tested by Wilcoxon rank-sum test.

Table S4. Beta diversity analysis of cecal microbiota (n = 8).

	binary_jaccard	bray_curtis	unweighted_unifrac	weighted_unifrac
Group R	0.67±0.02 [a]	0.67±0.03	0.59±0.22 [a]	0.31±0.02
Group S	0.58±0.02 [b]	0.62±0.02	0.54±0.04 [b]	0.27±0.01
p-value	0.002	0.073	0.039	0.070

Group R = the selected *S.enteritidis* resistant chicks; Group S = the selected *S.enteritidis*-susceptible chicks. Differences between two groups were tested by Wilcoxon rank-sum test. In the same colume, values with different letters are significantly different between two groups ($p < 0.05$).

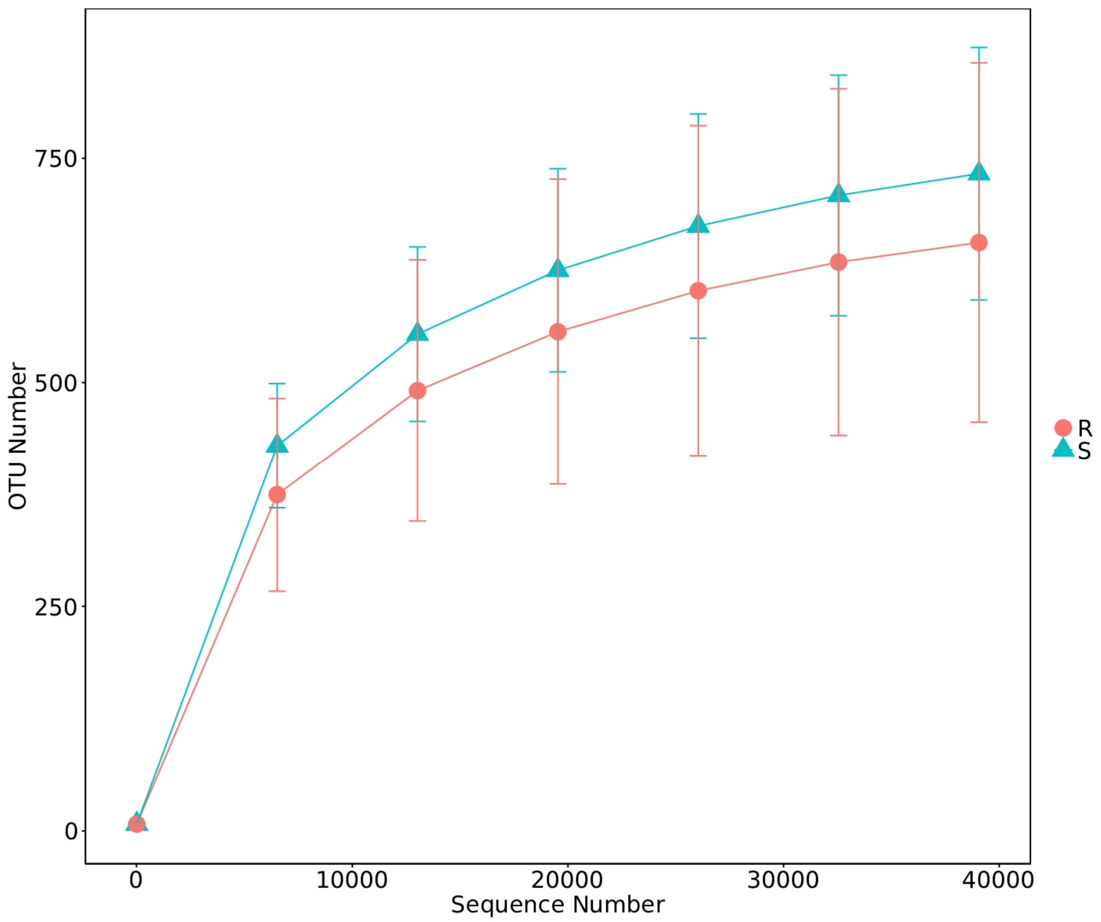

Figure S1. Rarefaction curves of the observed OTUs (n = 8). S = the selected *S.enteritidis*-susceptible chicks; R = the selected *S.enteritidis*-resistant chicks.
